# Excessive Venery, Masturbation, Continence, Etc.

**Published:** 1888-10

**Authors:** 


EXCESSIVE VENERY, MASTURBATION, CONTINENCE, ETC. By Joseph W. Howe, M. D. Pages 299; price, $2.75. New York: E. B. Treat & Co. 1888.

Dr. Howe's lectures treat of a subject upon which all physicians should be well posted, and also one upon which there should be no hesitation in speaking *plainly* to patients. This work is, therefore, worthy of careful attention.

